# Quantities of CD3+, CD8+ and CD56+ lymphocytes decline in breast cancer recurrences while CD4+ remain similar

**DOI:** 10.1186/s13000-022-01278-5

**Published:** 2023-01-10

**Authors:** Minna Mutka, Kristiina Joensuu, Mine Eray, Päivi Heikkilä

**Affiliations:** 1 Department of Pathology, HUSLAB, Helsinki University Hospital and University of Helsinki, FIN-00290 Helsinki, Finland; 2 University of Helsinki, FIN-00290 Helsinki, Finland

**Keywords:** Tumor infiltrating lymphocytes, Recurrence, Tumor stroma, Cancer immunoediting, Cancer immunoescape

## Abstract

**Background:**

Much is known about tumor infiltrating lymphocytes (Tils) in primary breast cancer, as this has been the focus of much research in recent years, but regarding recurrent breast cancer, only few studies have been done. Our aim was to compare the quantities of Tils in primary breast carcinomas and their corresponding recurrences and to analyze the differences in the tumor Tils compositions in correlations with recurrence-free times and the clinicopathology of the tumor.

**Methods:**

One hundred thirty-seven breast cancer patients self-paired for primary- tumor-recurrence were divided into three groups based on the length of the recurrence-free interval. H&E-staining and immunohistochemical staining with antiCD3, antiCD4, antiCD8 and antiCD56 were performed. Differences in Tils between primaries and recurrences, between the recurrence-free interval groups, and between different clinicopathologic parameters were statistically analyzed.

**Results:**

Fewer stromal CD3+, CD8+ and CD56+ lymphocytes were found at recurrences compared to the primaries. No significant change in the percentage of CD4+ stromal lymphocytes. ER-negative primaries, PR-negative or HER2-positive tumors had more Tils in some subgroups. Ductal primaries had more Tils than lobular primaries and G3 tumors had more Tils than lower-grade tumors. The corresponding differences at recurrences could either not be detected or they were reversed. The fastest recurring group had generally more Tils than the slower groups.

**Conclusions:**

CD4+ cell numbers did not decline from primary to recurrence in contrast to all other subclasses of lymphocytes. The proportion of CD4+ cells was higher in recurrences than in primaries. Tumors with a higher grade and proliferation rate had higher percentages of Tils. HER2+ and hormone receptor negative tumors tended to have higher Tils scores. In recurrences these differences were not seen or they were reversed.

## Background

Tumor-infiltrating lymphocytes (Tils) have become important prognostic and predictive factors in breast cancer in recent years. Increasing evidence suggest that high numbers of Tils predict a better prognosis in early-stage triple-negative (TN) and HER2+ breast cancer. In all breast cancer subtypes, greater quantities of Tils predict a greater likelihood of pathologic complete response in the neoadjuvant setting [1–4]. Tils also correlate with PD-L1 positivity and in TN- and HER2+ disease can even predict response for immune checkpoint inhibitors [5].

Although the evidence is overwhelming in early-stage breast cancer, less is known about the metastatic setting. A recent study showed that there are generally low numbers of Tils in the metastases of TN and HER2+ breast cancers. The same study showed that patients with a higher Tils score in their metastasis had a better prognosis, although in HER2+ tumors a higher Tils score was an adverse prognostic sign [6].

Immunohistochemical studies have shown lower numbers of Tils in the metastases of breast cancer compared to the corresponding primaries. One showed that CD8+ and CD20+ lymphocytes declined in metastases, in TN breast cancer even CD4+ and CD3+ lymphocytes declined [7]. Recently, two genomic studies reported a downshift in immunoactive-genes and an upregulation of immunosuppressive-genes in metastatic breast cancer [8, 9].

There are three phases of the cancer-immune system interaction in cancer immunoediting: elimination, equilibrium and escape. Cancer cells are eliminated in the first phase. In the second phase, there is equilibrium between the two. In the third phase, cancer cells escape active immunosurveillance [10, 11]. Tils are an integral part of this process and a comparison of them between primary tumors and metastases provides interesting information about the mechanisms of escape.

All published studies that compared Tils types and quantities in breast cancer in primaries and the corresponding metastases found that the counts of Tils decline in the metastatic setting. However, most studies have been small and some concentrated on only some molecular subtypes of breast cancer [7–9, 12–14]. Therefore, more information is needed to characterize Tils response in the progression of breast cancer. We studied the Tils scores, and specifically the numbers of CD3+, CD4+ and CD8+cells and NK-cells in a dataset comprising 137 primary tumor-recurrence pairs, which were divided into three groups based on their recurrence-free interval.

The first aim of this research was to compare quantities and subtypes of Tils in primary tumors and their corresponding recurrences. The second, was to compare Tils in different tumors grouped by recurrence-free interval. The third aim was to correlate Tils levels with tumor clinicopathologic factors.

## Methods

### Patients and tissue samples

The material comprised representative paraffin-embedded whole section tissue samples of primary tumors (PTs) and corresponding recurrences (Rs) of 137 patients, which had been collected from the archives of the Department of Pathology at the University Hospital of Helsinki [15]. Each patient was self-paired for PT and R. Recurrence was defined as any local or regional recurrence or any distant metastatic disease. The PTs had been operated between 1974-2006. Paired cases were divided into three groups according to the interval between PT and recurrence: group 1 short recurrence-interval (SRI, <2 years), group 2 intermediate recurrence-interval (IRI, 5-10 years) and group 3 long recurrence-interval (LRI, >10 years). All consecutive cases matching the required criteria were recruited. Detailed information about the cases is shown in Table 1 and a list of recurrence sites in Table 2.

**Table 1:** Clinocopathologic information of the cases

	**SRI**	**SRI %**	**IRI**	**IRI %**	**LRI**	**LRI %**
**Number of cases**	41		57		39	
**Age at diagnosis**						
<50	19	46.3	20	35.1	18	46.2
≥50	22	53.7	37	64.9	21	53.8
**Histologic type**						
ductal	24	58.5	36	63.2	16	41
lobular	17	41.5	19	33.3	23	59
other	0	0	1	1.8	0	0
**Grade**						
G1	4	9.8	7	12.3	8	20.5
G2	22	53.7	35	61.4	26	66.7
G3	15	36.6	15	26.3	5	12.8
**Estrogen receptor status**						
negative	18	46.2	18	32.1	12	31.6
positive	21	53.8	38	67.9	26	68.4
**Progesterone receptor status**						
negative	17	43.6	17	30.4	8	21.1
positive	22	56.4	39	69.6	30	78.9
**Ki67**						
≤20%	22	57.9	45	84.9	29	74.4
>20%	16	42.1	8	15.1	3	7.7
**HER2 status**						
negative	27	69.2	51	89.5	38	97.4
positive	12	30.8	5	10.5	1	2.6
**Size of primary tumor**						
≤20 mm	14	35	28	50	24	61.5
>20 mm	26	65	28	50	15	38.5
**Lymph node status at diagnosis**						
no metastasis	16	39	35	61.4	22	61.1
metastasis	25	61	22	38.6	14	38.9
**Tumour subtype**						
HR+HER2-	21	53.8	42	75.0	33	86.8
HR+HER2+	4	10.3	6	10.7	0	0
HR-HER2+	7	17.9	0	0	0	0
TNBC	7	17.9	8	14.3	5	13.2

*SRI* Short recurrence interval, <2 y, *IRI* Intermediate recurrence interval 5-10 y, *LRI* Long recurrence interval, >10 y. *HER2* Human epidermal growth factor receptor 2, *HR* Hormone receptor, *TNBC* Triple negative breast cancer

**Table 2:** Sites of recurrences

subcutaneous tissue	43
skin	27
soft tissue	23
bone	13
liver	7
lymph node	5
brain	4
lung	4
mesenterium	2
ovary	2
peritoneum	2
uterus	2
larynx	1
pleura	1
small bowel	1
Total	137

### Immunohistochemistry

Labvision immunostainer (Thermo scientific, Fremont, CA) was used to perform stainings for CD4, CD8, ER-alfa, PR-alfa and Ki-67. 4 μm sections were deparaffinised and pretreated in a PT module (LabVision UK Ltd., Suffolk, UK) in Tris-HCL buffer (pH 8.5). Endogenous peroxidase was blocked using hydrogen peroxidase. Primary antibodies were incubated at room temperature for 30 minutes with Dako RealEnVision/ HRP detection system (Dako, K5007), and the visualization of staining was done by REAL DAB+Chromogen (Dako, K5007) for 10 min.

Slides for CD3, CD56 and Her2 were stained in Ventana Benchmark Ultra (Roche, Tucson, AZ). Pretreatment was performed using Cell Conditioning Solution CC1 for 64 min at 98°C. The primary antibodies were incubated at 36°C for 32-48 min (Her2 48 min, CD56 32 min and CD3 40 min). OptiView DAB IHC Detection Kit (760-700 Ventana/Roche) was used for detection.

Finally, the slides, stained in both procedures described above, were counterstained with Mayer`s hematoxylin and mounted in a mounting medium.

The following antibodies were used: CD4 (dilution 1:500, clone 4B12, M7310, Dako), CD8 (dilution 1:100, clone C8/144B, M7103, Dako), CD3 (RTU, clone 2GV6, 790-4341, Ventana/Roche), CD56 (dilution 1:500, clone MRQ-42, 156R-96, Cell Marque), ER-alfa (dilution 1:50, clone 6F11, Novocastra), PR-alfa (dilution 1:100, clone 636, Dako), HER2 (dilution 1:400, clone CB11, Novocastra), and Ki67 (dilution 1:75, clone MIB1, DAKO).

All tumors with 2+ or more positivity in HER2 immunohistochemistry were tested for HER2 gene amplification with Inform HER2 Dual ISH in situ hybridization. The HER2 gene was targeted with a dinitrophenyl labeled probe and the chromosome 17 centromere was localized with a digoxigenin labeled probe (INFORM HER2 Dual ISH DNA Probe Cocktail, 780-4422, Roche/Ventana/Tuscon, AZ, USA 780–4422). HER2 was visualized as black signals with VENTANA ultraView Silver ISH DNP (SISH) Detection (760-098, Roche/Ventana/Tuscon, AZ, USA) and Chr17 as red signals with VENTANA ultraView Red ISH DIG Detection (780-4422, Roche/Ventana/Tuscon, AZ, USA).

**Fig. 1:**
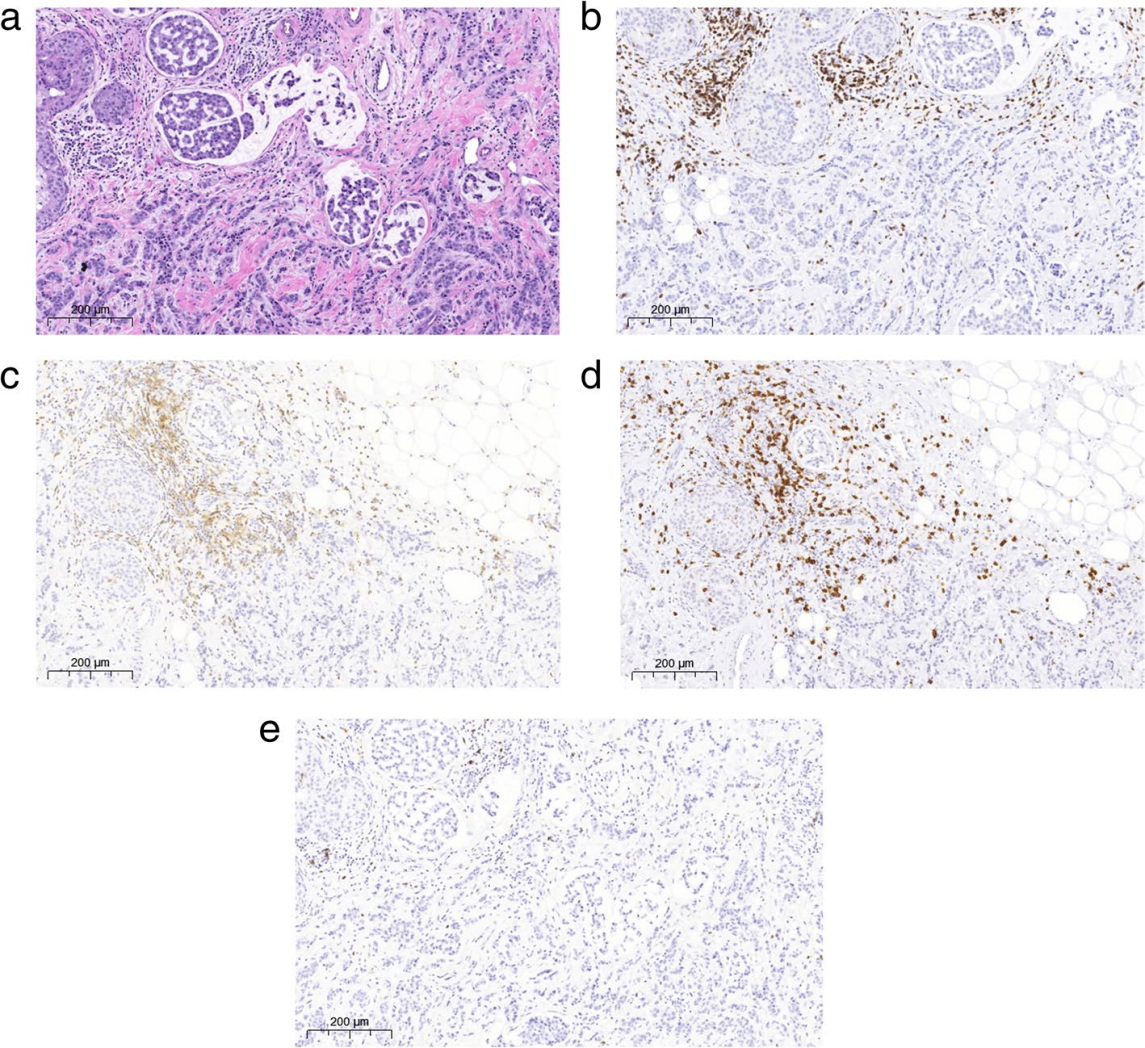
H&E stain and all immunohistochemical stainings of a PT (**a**) H&E (**b**) CD3 (**c**) CD4 (**d**) CD8 (**e**) CD56

**Fig. 2:**
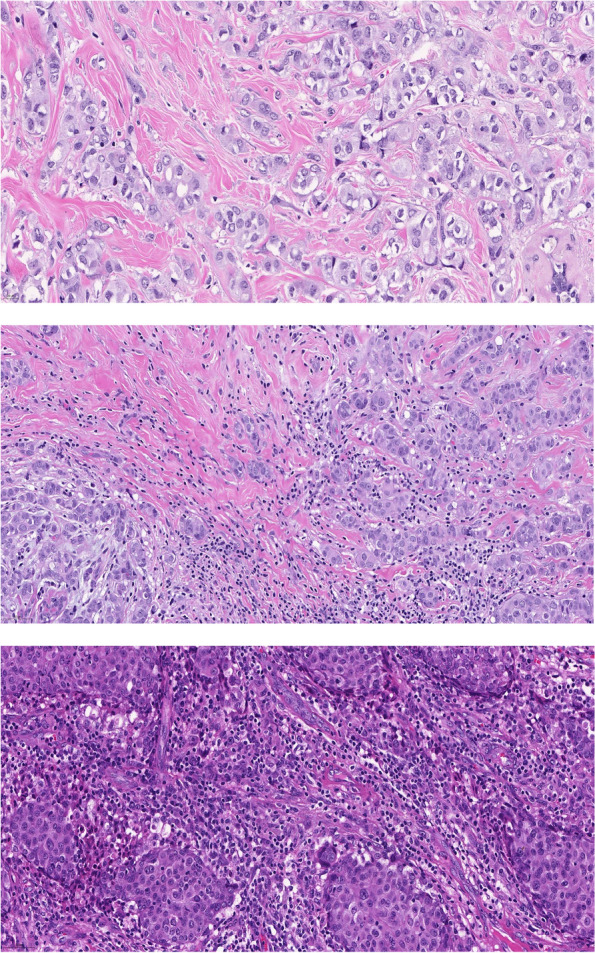
H&E stained sections of PTs (**a**) PT with Tils 1% (**b**) PT with Tils 10% (**c**) PT with Tils 30%

Hematoxylin & eosin sections and each immunohistochemical staining was evaluated according to the guidelines presented by the International TILs working group [16] and the International Immuno-Oncology Biomarkers Working Group [17]. The percentage of area occupied by lymphocytes in the overall area of the tumor stroma was assigned to H&E-stained sections and the percentage of area occupied by positive cells in the overall area to CD3-, CD8- and CD4 -stains in PTs and Rs (Figs. 1 and 2). The immunohistochemical percentages were often higher than the H&E percentage as lymphocytes were more easily detected with immunohistochemical stains. As the number of CD56-positive cells was generally very small, no percentage was given, but only the presence or absence of cells was recorded.

### Statistics

Statistical analyses were performed using SPSS 22.0 for Windows (SPSS Incorporation, Chicago, IL, USA). Differences between the expression of the markers in PTs and the corresponding Rs were tested using the paired samples t-test. Kruskal-Wallis test and Mann-Whitney U tests were used for comparing differences between the Groups and to correlate the Tils levels with clinicopathologic parameters. For estrogen receptor (ER) and progesterone receptor (PR) the cut-off point for positivity was 1%, and for Ki67 high >20%. Only the tumors with a positive HER2 gene amplification were considered to be HER2 positive. Probability values *p*<0.05 were considered significant.

## Results

### Tils percentages in H&E-stained sections decline between primaries and recurrences

**Fig. 3:**
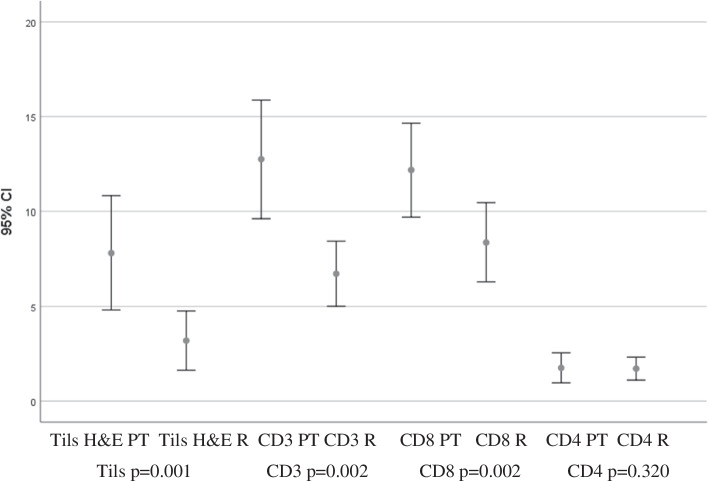
Comparison of tumor infiltrating lymphocytes in primaries and recurrences, mean and 95 % confidence interval, *p*-values for significance of difference. Figure created with SPSS 22.0 for Windows

There were significantly fewer Tils in the Rs compared to the PTs (*p*=0.001) (Figure 3).

The mean percentages of different subtypes of Tils in primaries and recurrences according to recurrence groups and clinicopathologic factors can be seen in Table 3.

**Table 3:** Mean Tils values according to different clinicopatologic factors  in primaries and in recurrences

	H&E primary	*p*-value	H&E recurrence	*p*-value	CD3 primary	*p*-value	CD3 recurrence	*p*-value	CD8 primary	*p*-value	CD8 recurrence	*p*-value	CD4 primary	*p*-value	CD4 recurrence	*p*-value
SRI	8.69	0.036*	3.62	0.463	13.03	0.081	9.04	0.865	13.89	0.619	9.78	0.346	3.46	0.01*	1.87	0.425
IRI	7.69		3.89		14.52		6.97		12.73		8.13		1.75		1.59	
LRI	4.35		2.12		8.85		5.9		10.56		7.19		1.4		2.08	
Ki67 ≤20%	7.40	0.298	3.12	0.671	11.50	0.104	7.09	0.423	12.01	0.164	8.22	0.964	1.91	0.024*	1.61	0.647
Ki67 >20%	6.38		3.38		15.00		6.47		13.87		7.13		2.42		1.68	
G1	6.07	0.001*	4.73	0.062	5.23	<0.001*	10.5	0.063	7.5	0.003*	11.79	0.068	1.56	0.585	3.06	0.225
G2	5.14		3.26		10.31		5.35		11.09		7.17		2.09		1.65	
G3	12.12		2.66		19.19		9.81		18.19		9.63		2.7		1.58	
Ductal	9.02	0.138	2.6	0.410	15.63	0.002*	7.11	0.793	14.36	0.126	7.97	0.917	2.61	0.422	1.37	0.118
Lobular	4.67		4.2		7.88		7.56		10.41		9.1		1.69		2.45	
ER negative	9.5	0.542	2.38	0.101	16.22	0.01*	5.94	0.089	14.27	0.787	6.2	0.046*	2.55	0.975	0.91	0.01*
ER positive	5.76		3.73		9.4		8.07		11.33		9.61		1.83		2.24	
PR negative	9.11	0.502	2.47	0.153	16.47	0.034*	6.13	0.095	14.79	0.293	6.73	0.281	2.94	0.811	0.95	0.063
PR positive	6.27		3.58		10		7.83		11.41		9.03		1.71		2.12	
HER2 negative	6.66	0.379	3.32	0.094	10.77	0.022*	7.3	0.779	11.31	0.034*	8.07	0.286	1.78	0.202	1.86	0.229
HER2 positive	9.94		2.46		21.64		7.0		19.07		9.85		3.76		0.87	
Size of primary <20 mm	7.42	0.140			12.2	0.13			12.61	0.362			1.98	0.100		
Size of primary ≥20 mm	6.86				12.14				12.67				2.35			

Mean % of different subtypes of Tils in primaries and recurrences according to recurrence groups and clinicopathologic factors and the *p*-values for the significance of the differences within factors. H&E Tils percentages in hematoxylin and eosin-stained sections, *SRI* Short recurrence interval <2 y, *IRI* Intermediate recurrence interval 5-10 y, *LRI* Long recurrence interval >10 y. *HER2* Human epidermal growth factor receptor 2, *ER* Estrogen receptor, *PR* Progesterone receptor, *G* Grade. Statistically significant differences within different clinicopathologic factors are marked with *

The Tils percentages of the PTs differed between the groups, the SRI group had the highest percentages and the LRI group had the lowest. In the pairwise comparisons, the difference was significant between the SRI and LRI groups (*p*=0.041). The percentages of the IRI group lay between the SRI and LRI groups, but the differences were not significant in the pairwise comparisons.

Percentages of Tils in the PTs differed depending on tumor grade. G3 tumors had the highest percentages and G2 the lowest. In the pairwise comparisons the differences in Tils were significant only between these two groups (*p*=0.001). G1 tumors had intermediate percentages of Tils, but the difference compared to the other two groups was not significant.

Differences in Tils percentages in the PTs did not depend on tumor histology, ER status, PR status, Ki67, HER2 status, size of PT or lymph node status at diagnosis.

### CD3+ lymphocytes are more abundant in primaries, higher grade, HER2 positive and hormone receptor negative tumors

There were significantly fewer CD3+ cells in the Rs compared to the PTs (*p*=0.002) (Fig. 3).

ER negative PTs had more CD3+ cells than ER positive PTs. PR negative PTs also had more CD3+ cells than PR positive PTs. No differences according to ER- and PR-status were seen in the Rs.

HER2 positive PTs had more CD3+ cells than negative PTs, but there were no differences in the Rs.

Ductal PTs had more CD3+ cells than lobular PTs, but there was no difference in CD3+ cells between these tumor types in the Rs.

G3 PTs had significantly more CD3+ cells than G2 and G1 PTs, in the pairwise comparisons the difference between G2 and G1 tumors was not significant, but for G2 and G3 (*p*=0.004) and G1 and G3 tumors (*p*<0.001) the differences were significant.

### Tumor grade and HER2 status correlate with larger numbers of CD8+ cells in primary tumors

**Fig. 4:**
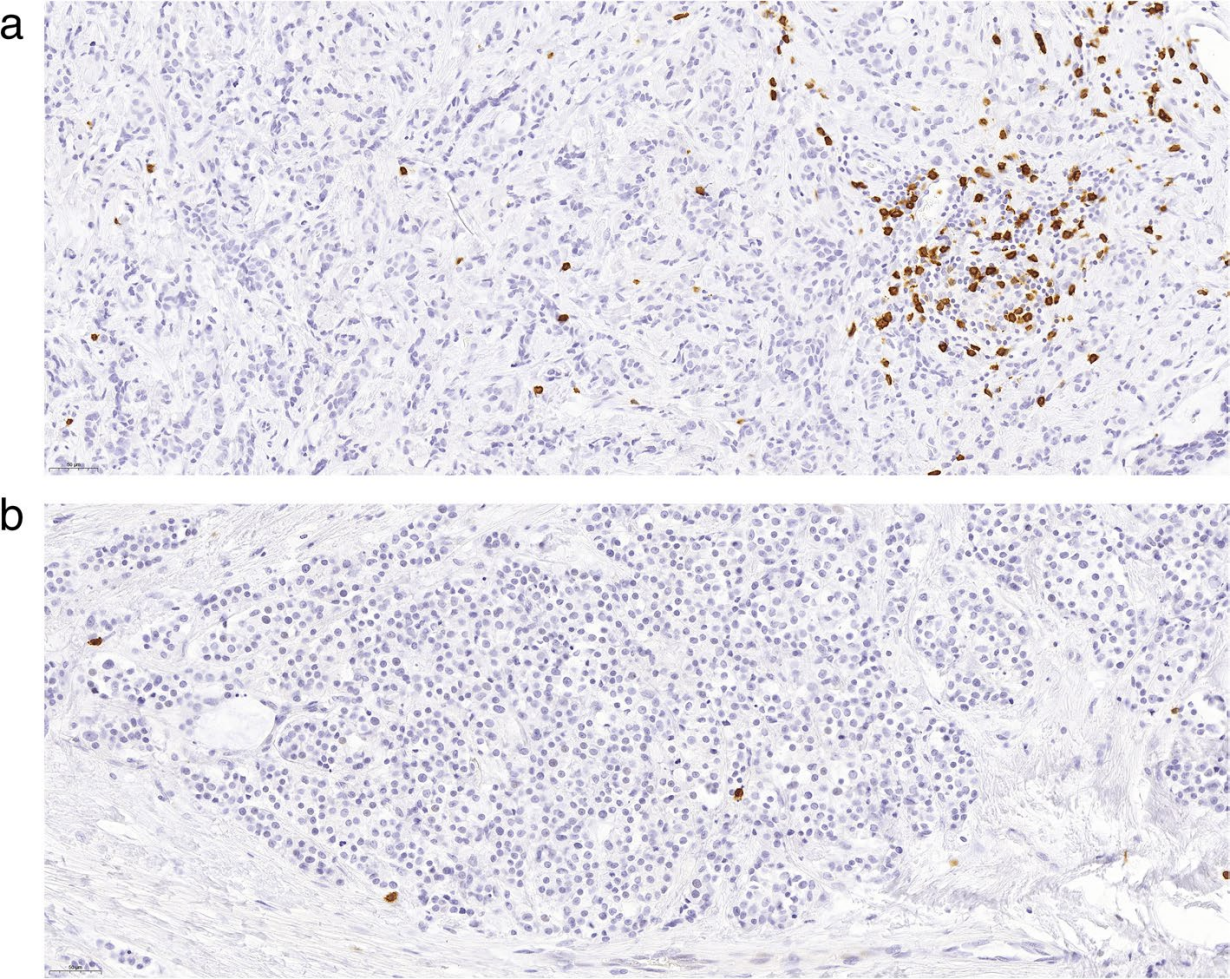
CD8-staining of a PT and the corresponding R (**a**) PT,CD8 percentage 10% (**b**) R, CD8 percentage 3%

There were significantly fewer CD8+ lymphocytes in the Rs compared to the PTs (*p*=0.002) (Figs. 3 and 4).

HER2 positive PTs had more CD8+ than HER2 negative PTs. This difference was not seen for the Rs.

G3 PTs had significantly more CD8+ cells than G2 and G1 PTs. The difference between G2 and G1 PTs in the pairwise comparisons was not significant. However, the differences were significant for G2 and G3 (*p*=0.016) and G1 and G3 (*p*=0.009) PTs. There were no significant differences according to grade in the Rs.

There were no significant differences in the percentages of CD8+ cells in the PTs between ER positive and ER negative and PR positive and PR negative tumors. However, ER-positive tumors in the Rs had more CD8+ cells. This same tendency was seen in PR positive and PR negative tumors but was not significant.

Tumor size, tumor histology, Ki67 status and lymph node status at diagnosis did not affect CD8+ cell counts.

### CD4+ cell numbers remain similar in primaries and recurrences

No significant difference in CD4+ cells occurred between the PTs and the Rs.

Significantly more CD4+ cells occurred in the PTs of the SRI group compared to the IRI group and LRI group, but there were no significant differences between the groups for the Rs.

Ki67- high PTs had more CD4+cells than Ki67- low PTs.

ER-positive tumors had more CD4+ cells in the Rs, but there was no difference in CD4+ cell counts between ER positive and ER negative tumors in the PTs.

No other clinicopathologic parameters led to significant differences in the percentages of CD4+ cells in the PTs or Rs.

### CD56+ cells are more often seen in primary tumors and highly proliferative tumors

50.5% of the PTs and 67% of the Rs had no CD56+ cells and 49.5% of the PTs and 33% of the Rs had positive cells. The difference between PTs and Rs is significant (*p*=0.005).

Fewer CD56+ cells occurred in the PTs in the LRI group compared to the SRI (*p*=0.003) and IRI groups (*p*=0.014).

There were more CD56+ cells in Ki67- high PTs (*p*=0.004).

No other significant differences were detected (Table 4).

**Table 4:** CD56+ cells in primaries and recurrences

	**CD56 PRIMARY**	**P**	**CD56 RECURRENCE**	**P**
**SRI**	79%	0.003*	38%	0.235
**IRI**	42%		39%	
**LRI**	34%		21%	
**KI67 ≤20%**	41%	0.004*	32%	0.834
**KI67>20%**	79%		32%	
**G1**	42%	0.105	21%	0.191
**G2**	42%		27%	
**G3**	67%		46%	
**DUCTAL**	47%	0.498	31%	0.705
**LOBULAR**	54%		36%	
**ER NEGATIVE**	56%	0.304	32%	0.984
**ER POSITIVE**	45%		32%	
**PR NEGATIVE**	63%	0.056	41%	0.208
**PR POSITIVE**	41%		28%	
**HER2 NEGATIVE**	49%	0.780	30%	0.204
**HER2 POSITIVE**	44%		50%	
**SIZE < 20 MM**	50%		33%	
**SIZE ≥ 20 MM**	50%	1.000	33%	1.000

Percentages of tumors with CD56+cells and *p*-values for significance of differences, *SRI* Short recurrence interval <2 y, *IRI* Intermediate recurrence interval 5-10 y, *LRI* Long recurrence interval >10 y. *HER2* Human epidermal growth factor receptor 2, *ER* Estrogen receptor, *PR* Progesterone receptor, *G* Grade. Statistically significant differences within different clinicopathologic factors are marked with *

## Discussion

To our knowledge, there are nine studies comparing Tils or factors associated to Tils in breast cancer PTs and Rs [6–9, 12–14, 18, 19]. Three of the studies considered only a few forms of breast cancer [6, 14, 18] and two had limited the sites of metastases [13, 14]. These studies have very limited numbers of pairs, ranging from 15 [7, 18] to 87 [12]. Our study comprises 137 pairs, compares several types of breast cancer, does not limit the sites of metastases and is also the only study that considers CD56+ NK cells and the recurrence-free interval.

Our results confirmed those of other primary-metastasis self-paired-sample studies, which showed there were generally lower numbers of lymphocytes in Rs than in the PTs [7–9, 12–14]. We saw this decline in the percentages of lymphocytes in H&E-stained sections and in CD3+, CD8+ and CD56+ cells, but interestingly, not in CD4+ cells.

CD8+ T-lymphocytes are cytotoxic cells that destroy tumor cells by inducing cytolysis with the granzyme B-perforin complex [4]. These cells are important in the initial elimination phase of the immunoediting cycle, therefore to survive and spread, cancer cells must overcome the effects of these cells. Consequently, CD8+ cells have been reported to be a good prognostic sign especially in TN and HER2 breast cancer [20–22], and they have consistently been found to be decreased in metastasized breast cancer [7, 12–14]. The same is true for CD56+ NK cells, although the significance of these cells is less explored and less clear [23, 24].

The role of CD4+ T-lymphocytes is dual. CD4+ helper-T-lymphocytes of the Th1 type activate CD8+ cells, which is mediated by the secretion of activating cytokines such as IFNγ and by direct cell-to-cell contact. On the other hand, CD4+ helper-T-lymphocytes of the Th2 type induce an immunosuppressive reaction. There are also CD4+ regulatory-T-lymphocytes (Tregs) that express FOXP3 and are generally immunosuppressive [25, 26], these have been found to be an adverse prognostic sign in many but not all studies [27–30], as the evidence is still conflicting [31]. CD4+ cells in general have been associated with a worse prognosis in some studies [32, 33], and a better prognosis in another [26]. A high CD4+/CD8+ ratio is associated with a worse prognosis [34]. In the present study, we found no significant difference in the quantities of CD4+ cells between PTs and Rs. The numbers of CD4+ cells did not diminish, whereas CD3+, CD8+ and CD56+ cell numbers declined. This suggests that the proportion of CD4+ cells rose, which would imply that these cells have an important role in cancer escape from the immune system.

In the initial phase of an inflammation, there is a balance between Th1 T helper cells and Th2 T helper cells. This balance is broken in cancer immunescape, as the function and amount of Th1 cells are decreased. Why this happens is unknown, but this may in part be due to CD4+ Tregs. The function of Tregs is thought to be to protect the body from excess inflammation. In the prolonged inflammation associated with breast cancer the appearance of Tregs is a harmful process, as limiting the inflammation helps the cancer cells escape from the immune system [34].

Changes in the tumor cells may also affect T cell function. With time tumor cells accumulate genetic and epigenetic changes that may alter their sensitivity to the immune system. Some cancer cells lose their antigens or MHC-molecules making them harder for the immune system to recognize. Cancer cells can also become insensitive to T cells if they lose their apoptotic mechanisms. Some cancer cells can even secrete cytokines that activate harmful Tregs [35].

Cancer cells can express PD-L1, which has been proven to alter the immunreaction to cancer cells [36]. The PD-L1/PD1 interaction leads to apoptosis of tumor-spesific T cells, which could explain the lower amounts of certain T cells in Rs. This interaction also inhibits T cell proliferation and function. At the same time, it helps CD4+ cells to differentiate into FOXP3+ Tregs [37]. This has therapeutic implications as PD-L1/PD-1 blocking agents are now being used in breast cancer therapy.

Tils can predict treatment response and response to immune checkpoint inhibitor therapy. Therefore, the changes in Tils amounts as the cancer progresses can also affect treatment options and treatment response [5].

We found that G3 tumors had more Tils of all subclasses than lower grade tumors. Also, faster proliferating tumors had more CD4+ cells and CD56+ cells than slowly proliferating tumors. This agrees with previous findings in which, more aggressive tumors evoked a stronger immune reaction [38]. This might be due to either higher numbers of mutations that lead to immunogenic neoantigens or to more cell death in highly proliferative tumors that lead to more antigen presentation and stress proteins [39].

The samples in this study were divided into three groups (SRI, IRI and LRI) according to the recurrence-time interval. We found that the Tils percentages as assessed in H&E-stained sections were higher in the SRI group than the IRI and LRI and a similar difference was found for CD4+ cells. However, this difference was not significant for CD3+ and CD8+ cells. This would imply that the proportion of CD4+ cells is, ceteris paribus, higher in the fastest-recurring group. These findings also suggest that CD4+ cells are essential in cancer progression and cancer immunoescape.

Higher numbers of Tils in more aggressive tumors is consistent with the fastest-recurring tumors having more Tils, as more aggressive tumors tend to recur earlier. The relatively higher numbers of CD4+ cells in the fastest recurring group might therefore mirror enhanced immune reaction in these tumors and be associated with Th1 CD4+ cells. However, the number of CD4+ cells with immunosuppressive functions might already have become elevated in these tumors as early as the primary stage, which might be an early sign of impending or actual immune escape. Therefore, further research is needed to understand better the function of CD4+ cells and their role in cancer immune escape.

Ductal carcinomas had more CD3+ cells than the lobular carcinomas, a previous study also reported more Tils in ductal carcinomas than lobular carcinomas [40]. HER2 negative carcinomas had fewer Tils than HER2 positive carcinomas [38], but these differences were not significant for all Tils subclasses. Although, many differences were seen between the groups and between different clinicopathologic parameters in the PTs, these differences were not seen in the Rs.

ER- and PR-negative PTs had more Tils than ER- and PR-positive PTs. Interestingly, in Rs CD8+ and CD4+ percentages in the ER-positive tumors seemed to be significantly higher than in negative tumors: contrary to that found in the PTs. A similar, but not significant, shift was seen in CD3+ and H&E Tils percentages, and also in PR positive versus negative tumors. The finding that Tils counts in ER-positive breast cancer did not change significantly in the recurrence, implies that immune escape has little importance in this tumor type and that the immune system might be malfunctioning. Indeed, it is known that ER has many immunosuppressive functions [41].

The main limitation of this study is the subjective nature of the visual assessment of Til percentages. However, this was lessened by the assessments made by several pathologists. In the future, digital solutions might reduce this problem. Comparisons of Tils according to different clinicopathologic parameters within recurrence groups were on occasion limited by the small numbers of different types of tumors per recurrence group: especially for the rarer tumors such as TN breast cancer.

## Conclusions

This is hitherto the largest published study that investigates tumor infiltrating lymphocytes in paired primary-recurrence breast cancer cases. We showed that the Rs had significantly fewer CD3+, CD8+ and CD56+ lymphocytes than the PTs, but that the amounts of CD4+ cells remained similar in the PTs and the Rs, suggesting that CD4+ cells could play an important role in cancer immunoescape. We also found many interesting differences in Tils counts in PTs depending on different cilinicopathologic findings, interestingly these differences were not seen in the Rs.

## Data Availability

The datasets generated during and/or analysed during the current study are available from the corresponding author on reasonable request.

## Abbreviations

Tils: Tumor infiltrating lymphocytes
ER: Estrogen receptor
PR: Progesterone receptor
HER2: Human epidermal growth factor receptor 2
TN: Triple negative
NK-cells: Natural killer cells
PT: Primary tumor
R: Recurrence
SRI: Short recurrence interval
IRI: Intermediate recurrence interval
LRI: Long recurrence interval
G: Grade
Treg: Regulatory T lymphocyte
